# Random-telegraph-noise-enabled true random number generator for hardware security

**DOI:** 10.1038/s41598-020-74351-y

**Published:** 2020-10-14

**Authors:** James Brown, Jian Fu Zhang, Bo Zhou, Mehzabeen Mehedi, Pedro Freitas, John Marsland, Zhigang Ji

**Affiliations:** 1 School of Engineering, Liverpool John Moores University, Liverpool, L3 3AF UK; 2 National Key Laboratory of Science and Technology on Micro/Nano Fabrication, Shanghai Jiao Tong University, Shanghai, 200240 China

**Keywords:** Electrical and electronic engineering, Electronic devices, Computer science, Characterization and analytical techniques

## Abstract

The future security of Internet of Things is a key concern in the cyber-security field. One of the key issues is the ability to generate random numbers with strict power and area constrains. “True Random Number Generators” have been presented as a potential solution to this problem but improvements in output bit rate, power consumption, and design complexity must be made. In this work we present a novel and experimentally verified “True Random Number Generator” that uses exclusively conventional CMOS technology as well as offering key improvements over previous designs in complexity, output bitrate, and power consumption. It uses the inherent randomness of telegraph noise in the channel current of a single CMOS transistor as an entropy source. For the first time multi-level and abnormal telegraph noise can be utilised, which greatly reduces device selectivity and offers much greater bitrates. The design is verified using a breadboard and FPGA proof of concept circuit and passes all 15 of the NIST randomness tests without any need for post-processing of the generated bitstream. The design also shows resilience against machine learning attacks performed by the LSTM neural network.

## Introduction

Cyber-security is a key concern and grows in importance as the Internet of things (IoT) takes root, where billions of devices will be connected across the globe^1^. The necessity to secure all of these devices requires novel approaches to traditional problems in the cyber-security domain as existing techniques are not viable for the new challenges presented by many of these IoT devices^2^. One such problem is the need for random numbers which are essential for secure communication between devices^3^. Traditional methods for random number creation rely upon harvesting entropy from some environmental factor such as circuit noise or user input and use this as a seed for a pseudo-random number algorithm^4^. These traditional methods are now under huge strain as the ultra-low power and device area in typical IoT edge units don’t possess the computing power required by the traditional methods. This is also compounded by another problem where machine learning attacks are now able to crack and predict the output from the traditional pseudo-random algorithms^5^. A potential solution to this problem is to directly extract entropy from a natural phenomenon and converts it to random numbers. This type of design is commonly referred to as “True Random Number Generator” (TRNG), although it should point out that no amount of statistical analysis can confirm that a generator is truly random. Many TRNG designs have been proposed that make use of a wide range of entropy sources, but none have yet been widely accepted as a solution for security concerns. One of the first entropy sources was thermal noise either extracted directly from a resistor^6^ or through its effect on digital circuits such as the introduction of clock-jitter or meta-stability^7–22^. The weaknesses of the thermal noise-based designs are its sensitivity to noise-based attacks, compromising the randomness of the output, as well as issues with the design complexity required for post-processing and tuning of the system^10,23–26^. Many other entropy sources have been used for TRNGs, such as the timing of time dependent dielectric breakdown (TDDB)^27^, Photonics^28–31^, and current variation or switching time of emerging technologies such as ReRAM^32–34^. TDDB-based designs suffer from high complexity of circuit design and processing circuits which limit their use in low-area, low-power applications. The efficient and secure quantum based TRNGs have been demonstrated^35^, but different applications will use different types of TRNGs in the future.

Another entropy source that has shown promise is random telegraph noise (RTN). RTN is a phenomenon observed in scaled CMOS devices caused by the capture and emission of charge carriers from the conduction channel to traps within the gate dielectric that results in variation of the threshold voltage and channel current over time^36^. The variation in the capture and emission time can be extracted and used as an entropy source. Several RTN-based TRNG designs have been proposed, however all have drawbacks of low speed, complex tuning requirements, complex post-processing, vulnerability to over-sampling attacks, or the need for large device arrays to find a device with suitable RTN to be used as an entropy source^37–40^.

In this work, we present a novel TRNG design using a new approach to RTN extraction and processing which overcomes the weaknesses of RTN based TRNGs mentioned above. The proposed design makes use of a new edge-to-pulse scheme which enables, for the first time, multi-level and abnormal RTN to be used to increase speed and to reduce device selectivity. We also use a new processing technique where the pulses from the edge-to-pulse circuit are used to sample an oscillator, removing the need for complex post-processing and for tuning of the sampling rate, as well as increasing resistance to over-sampling attacks. The proposed design passes the 15 NIST Special Publication 800-22 randomness tests^41^ which is the industry standard tests for TRNG randomness. The whole design is verified experimentally, with a proof of concept circuit using a CMOS transistor as the entropy source, the edge-to-pulse circuit built on breadboard, and the processing circuit implemented on an FPGA.

## Results

### RTN in nano-scale CMOS transistors

**Figure 1:**
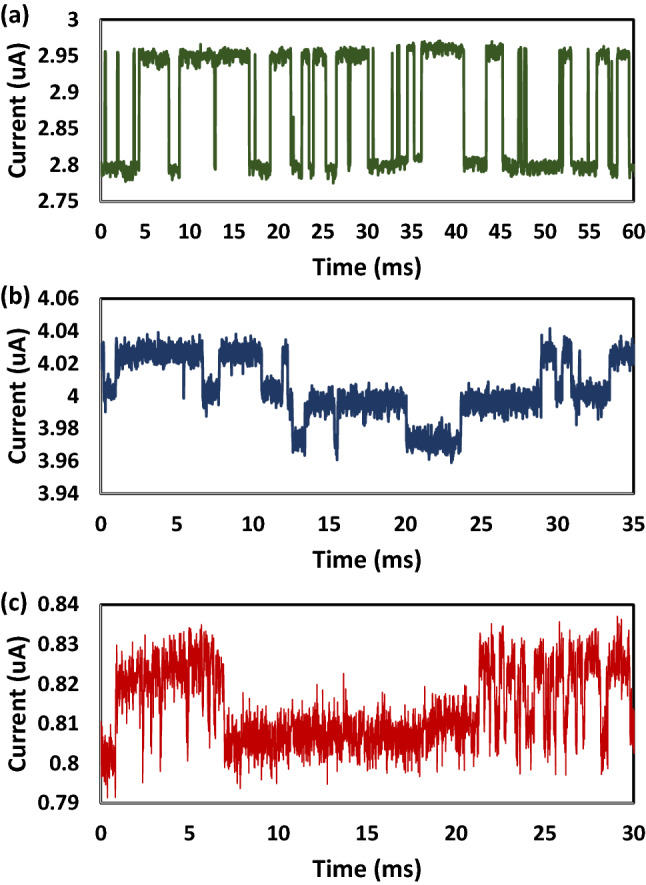
Major different types of RTN observed in measurement of individual transistors of the same design and size. (**a**) An example of two-level RTN, the most well studied type of RTN utilised by traditional RTN based TRNGs. Two-level RTN is caused by a single dominating trap within the measured device. (**b**) An example of multi-level RTN, a more complex RTN signal which cannot be used by traditional RTN based TRNGs but can be used by our newly presented TRNG design. Multi-level RTN is caused by multiple active traps being present within the measured device. (**c**) An example of abnormal RTN, the most complex type of RTN discussed here. Abnormal RTN could not be used in traditional designs and could cause major issues such as oversampling. With our new design abnormal RTN can be used without issue. Abnormal RTN is caused by dominating traps causing other traps to be obscured when the dominating trap is in one particular state or another.

RTN is caused by traps, which are the electrical-active defects within the gate dielectric of nano-scaled devices. These defects can be formed during fabrication of the device or generated through electrical and thermal stressing^42^. Previously, when researchers explored RTN as an entropy source for a TRNG, only two-level RTN is considered. This type of RTN is shown in Fig. 1a where a constant gate (Vg) and drain (Vd) voltage are applied and two clear levels can be observed in the measured drain current (Id). However, it is often the case that more than one trap is present in a given device, resulting in an RTN signal with more than 2 visible levels, This is referred to as multi-level RTN^43^ and one example is given in Fig. 1b, where we can observe 4 clear levels in the Id. When 4 clear levels can be observed, there are 2 active traps influencing the channel current. It is possible to have more traps active, although in nano-scale devices it is rare to have more than a few clearly discernible traps. The number of traps present is proportional to device area and the single trap impact is inversely proportional to it^44,45^. It is also often observed that RTN is not always consistent where some traps may only be active when a dominating trap is in a certain state^46^. This can result in Id measurements like or similar to Fig. 1c where there is clearly a slower trap and a faster trap, but the faster trap is only active when the slow trap is in the empty state.

### TRNG design utilising multi-level RTN

The TRNG presented in this work has three major parts. The first part is the entropy source which is a single nano-scale transistor with a constant Vg and Vd applied to it, whose Id current is amplified so that it can be used as an input to the edge-to-pulse circuit. The second major section of the TRNG circuit is the edge-to-pulse circuit which is used to perform the analogue to digital conversion where the amplified RTN signal is converted to digital pulses that can be used as an input to the processing circuit. The final part of the circuit is the processing circuit, which converts the digital pulses to random numbers through the sampling of a high frequency oscillator. The processing circuit also acts as a bridge between the asynchronous random number generation and any synchronous circuit it is attached to.

### Edge-to-Pulse circuit

The edge-to-pulse circuit takes an amplified RTN signal and converts the trapping and de-trapping events into short pulses. These short pulses can then be used by the processing circuit to generate random numbers based upon the timing interval of these events. The edge-to-pulse circuit consists of three sub-circuits.

**Figure 2:**
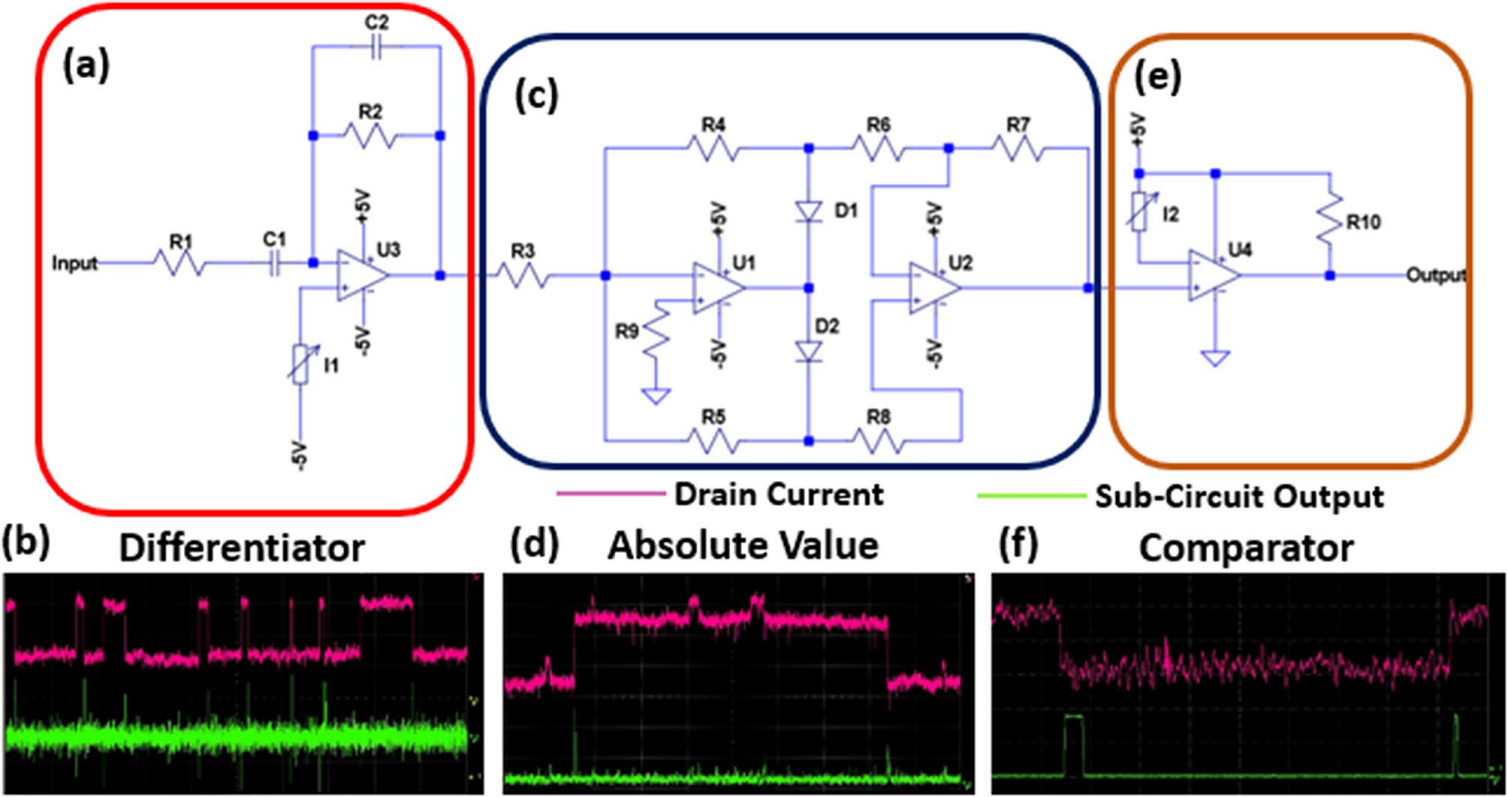
Edge-to-pulse circuit diagram and example outputs. (**a**) Circuit diagram of differentiator used to convert RTN edges to voltage spikes. (**b**) Example output signal from differentiator for the given input RTN signal, edges converted to positive and negative spikes. (**c**) Circuit diagram of absolute value circuit used to convert positive and negative differentiator spikes to all positive spikes. (**d**) Example output signal from absolute value circuit for the given RTN input. All voltage spikes are now positive. (**e**) Circuit diagram of comparator used to convert voltage spikes from absolute value circuit to digital pulses that can be used as input to the FPGA. (**f**) Example output from comparator circuit for given RTN input, spike has been converted to square pulse.

The first of these sub-circuits is a differentiator that converts the RTN trapping and de-trapping events into positive and negative voltage spikes respectively as seen in Fig. 2a,b. It is important that the differentiator circuit is biased so that no edges give a constant output voltage of 0. This 0 V no edge output is set so that differentiated de-trapping events will give a negative voltage spike which is required for the next absolute value sub-circuit. In our proof of concept circuit, this 0 V bias was achieved with a variable resistor connected to the non-inverting terminal supplied with − 5 V. In a commercial product this would be replaced with a fixed resistor as the expected Id current from the entropy source for the given Vg and Vd would be known.

The second sub-circuit is the absolute value circuit seen in Fig. 2c,d. It converts the negative spikes from the differentiated de-trapping events into positive spikes so that they can be digitised by the same comparator. The gain of the absolute value circuit can be greater than 1 if required, but in our experimental case it is left at unity as no further amplification was required for the spikes to be of sufficient magnitude as input for the comparator.

**Figure 3:**
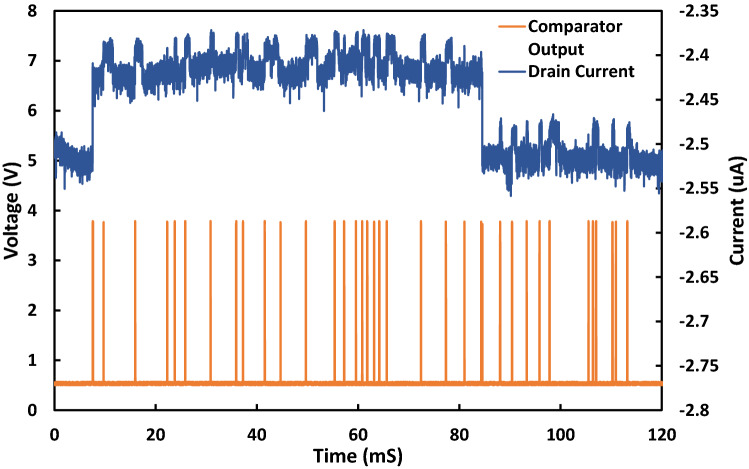
Output of comparator for given RTN input signal to edge-to-pulse module. Output of comparator has a magnitude of 3 V making it a suitable input for the FPGA. The comparator output is triggered on both the edges of the large slower trap and the smaller faster trap, showing our design can make use of multi-level RTN. Some of the edges of the small trap can be missed, however, as the magnitude of the trap is very close to the background noise.

**Figure 4:**
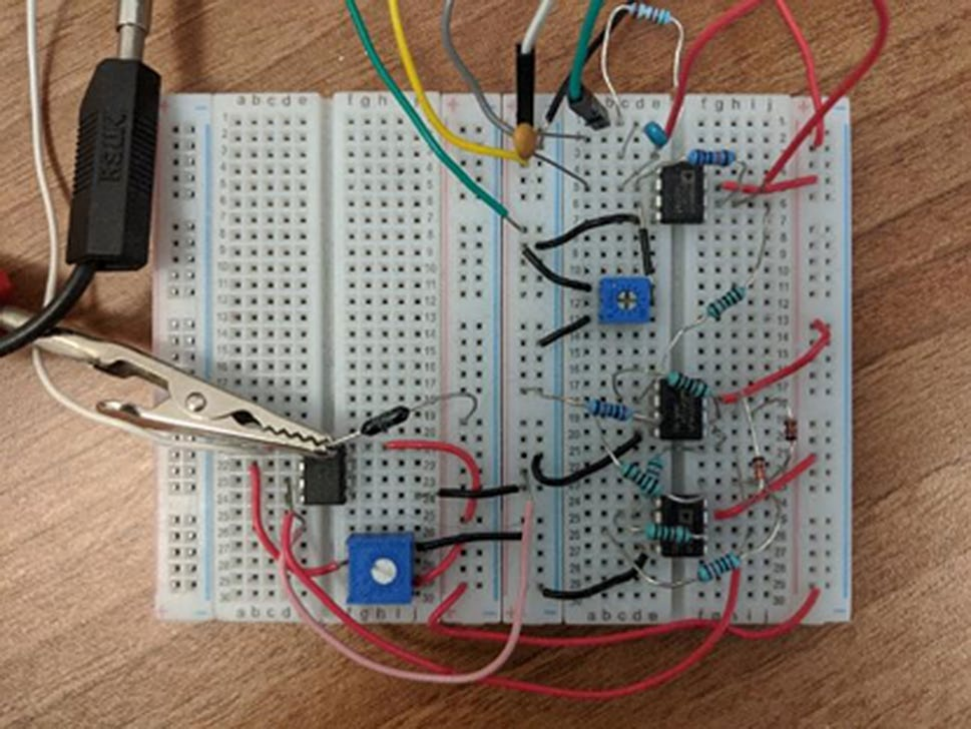
Photograph of Edge-to-pulse circuit, implemented on breadboard, including differentiator, absolute value, and comparator circuits. Amplified RTN signal is used as input to this circuit and the comparator output is sent to processing FPGA.

The final sub-circuit shown in Fig. 2e,f is a comparator which is used to convert the analogue spikes from the absolute value circuit to digital pulses which can be used as an input to the processing section of the TRNG. Figure 3 shows the output with a multi-level RTN input. The comparator is set to give a 0 V or 3 V output for low and high levels which is the required by the I/O of the FPGA used for the processing section. The reference voltage for the comparator input is set to be slightly higher than the background noise of the absolute value circuit so that all RTN event spikes will be digitised. The complete breadboard implementation of the edge-to-pulse circuit is shown in Fig. 4.

### Processing circuit implemented on a FPGA

**Figure 5:**
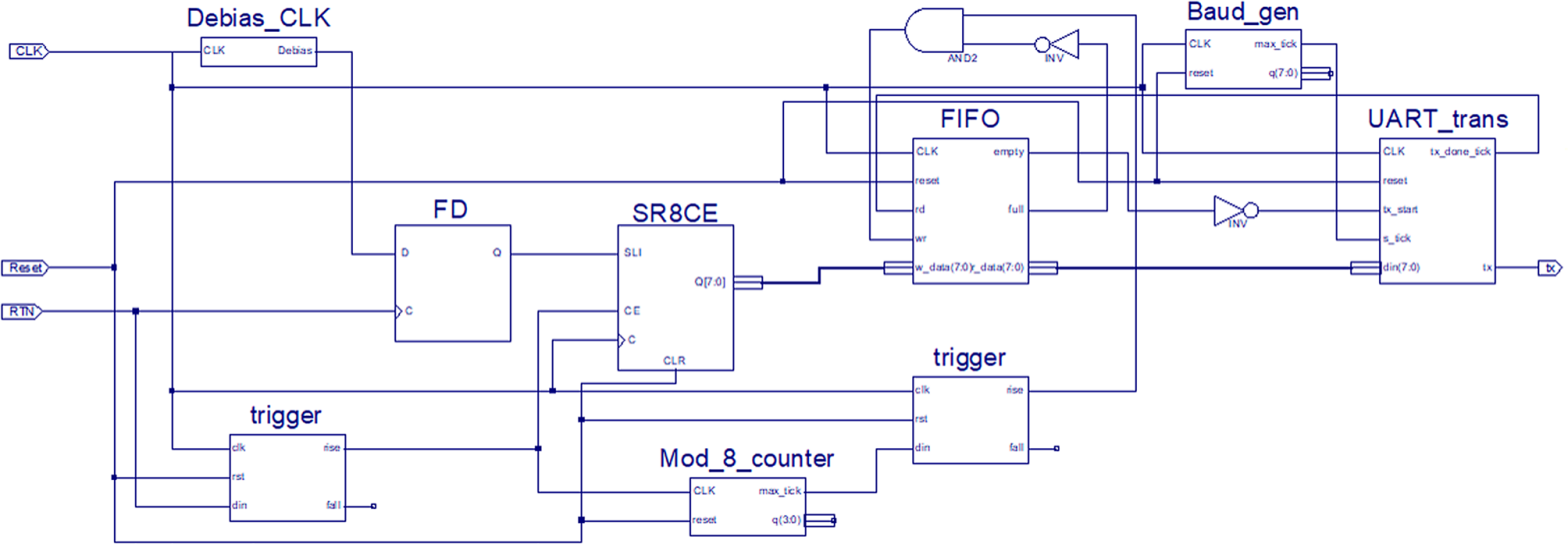
Block diagram of FPGA section of the proof of concept TRNG. This block diagram was used for the visual programming part of the FPGA implementation. Each block is created using VHDL programming, with the Debias and Trigger modules being custom code and the other modules created using conventional designs. This block diagram shows all 3 key parts of the FPGA design, which are the sampling, buffering, and transmission circuits.

The second major part of our TRNG design is the processing circuit where the digitised pulses from the edge-to-pulse circuit are used to generate random numbers, the complete block diagram of this part of the TRNG is shown in Fig. 5. The processing circuit itself can be split into three sub-circuits. The first two sub-circuits for sampling and data buffering are essential for random number generation. The third sub-circuit is used for transmission of the generated numbers to an external analysis PC which is used for result verification. To perform the data transmission, the RS232 UART communication on the FPGA development board was used. The requirements of the RS232 communication governed the selection of the 8-bit words of random numbers that were created in the first two sub-circuits, as well as the maximum data transfer rate of 19,200 bps, which is limited by the maximum clock speed of the FPGA. Without the limitations of the RS232 communication these variables could be changed to fit the requirements of the given application.

The sampling sub-circuit contains two key components, the first of these is a D-Flip-flop which is used to asynchronously sample an oscillator (in this case the clock of the FPGA) whenever a rising pulse edge is received from the edge-to-pulse circuit. To achieve the sampling, the oscillator is connected to the D-input of the flip-flop and the output of the edge-to-pulse circuit is connected to the clock input of the flip-flop. The output ‘Q’ will update to whatever value the oscillator was at when the rising edge of the pulse was received. The second key component in this section is a trigger module that is used to ensure each pulse from the edge-to-pulse circuit only results in one value being written into the data buffering section. This is achieved by the trigger module having both the FPGA clock and the edge-to-pulse output connected as inputs. Every time a pulse rising edge is received from the edge-to pulse circuit the trigger unit will output a pulse that has a duration of a single clock cycle of the FPGA clock. This single clock cycle pulse can then be used as an enable signal in other parts of the circuit that are required to be synchronous.

The data buffering sub-circuit is used to buffer the random numbers that are generated at an asynchronous non-constant rate so that they can be read at a constant synchronous rate. In our proof of concept circuit, it also converts the single generated random bits into 8-bit random words so they can be transmitted following the RS232 communication scheme. The first important module of the data buffering sub-circuit is the serial-to-parallel converter. For the proof of concept circuit this module converts eight serial input random numbers to a single parallel 8-bit random word which can then be sent to the first in first out (FIFO) buffer. The second key module is a counter which outputs a single pulse for every eight input pulses from the trigger module of the sampling sub-circuit. This counter is used to control the transmission of data from the serial to parallel converter to the FIFO buffer. The data buffering sub-circuit also contains a trigger module that is the same as in the sampling sub-circuit. This second trigger module is used to ensure that the output from the counter is a pulse that only lasts for a single clock pulse of the FPGA. This is important to ensure that when data is transferred from the serial to parallel converter and then to the FIFO buffer, it is only transferred once per 8-bit word.

The final key module in the data buffering sub-circuit is the FIFO buffer, the main purpose of this module is to allow the random words to be read from the TRNG at a different rate from that which they are generated at. This concept is key as the random bits are generated at the rate of the RTN events whereas any application using these numbers can use them at a rate dictated by the application’s requirements. The depth of the FIFO buffer can also be adjusted depending on the application’s requirements. The FIFO buffer will write in the random number sent from the serial to parallel converter every time an enable pulse is received from the data buffering trigger module. When the FIFO is full, data won’t be written in and instead will be discarded. The FIFO buffer will output a random 8-bit word every time an enable signal is received from the transmission sub-circuit unless the FIFO buffer is empty in which case nothing will be transmitted.

The transmission sub circuit is used to transmit the random 8-bit words from the data buffering sub-circuit to an external analysis PC. In practice this sub-circuit is not required for TRNG operation, but it is used here in our proof of concept circuit so analysis such as the NIST tests could be performed. The first key module in the data transmission circuit is the baud generator, which divides the FPGA clock signal down by 16 times to give a baud rate for the RS232 UART communication. The second key module is the UART transmitter, this is a module that follows the RS232 communication scheme and has the maximum data transfer rate is 19,200 bps for the used FPGA. This maximum data transfer rate is the bottleneck for our proof of concept circuit. The TRNG itself can generate random bits at much faster rate, the maximum of which is limited by the slew rate of the op-amps in the edge-to-pulse circuit.

### Sampling oscillator parameters

The parameters for the oscillator are key for the proposed TRNG to work properly and output unbiased random numbers. The key parameters are the frequency of oscillation, and duty factor.

**Figure 6:**
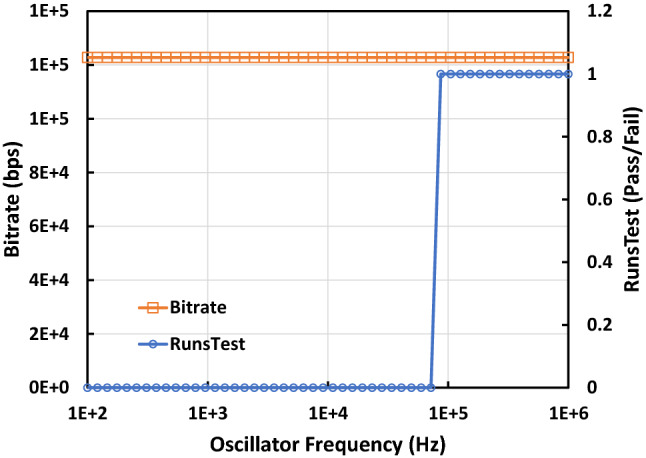
Output bitrate and Runs Test result against the frequency of the oscillator being sampled by the RTN edge pulses. The Runs Test is used to find runs of ‘1’ or ‘0’ and determines whether a run of the same result that number of times is acceptable in a bitstream of the given length, based on a null hypothesis theory with a significance value of 0.01. The Runs Test gives a result of ‘1’ if it passes and a ‘0’ if it fails, a fail indicates oversampling of the oscillator by the RTN signal causing long chains of the same result. The RTN signal simulated for this test uses 5 traps with τc and τe values extracted from characterisation of real traps. Both the rising and trailing edges of the RTN signal were used for sampling giving and Fosc_min_ of 1.21E + 5 Hz. The minimum frequency that could pass the Runs Test was 8.6E + 4 Hz and the output bitrate was fixed at 122,800 bps.

For the frequency of oscillation, the key point is that the frequency should be high enough that the signal is not sampled multiple times by the RTN before switching state. If the oscillator is too slow and sampled multiple times before switching, the output will contain long chains of the same bit which lowers entropy and causes the bitstream to be non-random. Figure 6 shows a simulation of a TRNG with multiple RTN traps with fixed capture times (τc) and emission times (τe) operating across a range of oscillator frequencies. The bitrate remains almost constant as this is controlled by the RTN event timing. When the oscillator frequency is reduced below Fosc_min_, however, the generated bits start to fail the Runs Test for randomness. Fosc_min_ is defined by Eq. (1) where n is the number of traps present in the entropy source. To act as a rule of thumb when choosing an oscillator frequency, it should be selected to be as high as possible without violating the other key parameters as any frequency above Fosc_min_ will give the same results.

1 $${Fosc}_{min}=\sum_{i=1}^{n}\frac{2}{{\tau c}_{i}+{\tau e}_{i}}.$$

**Figure 7:**
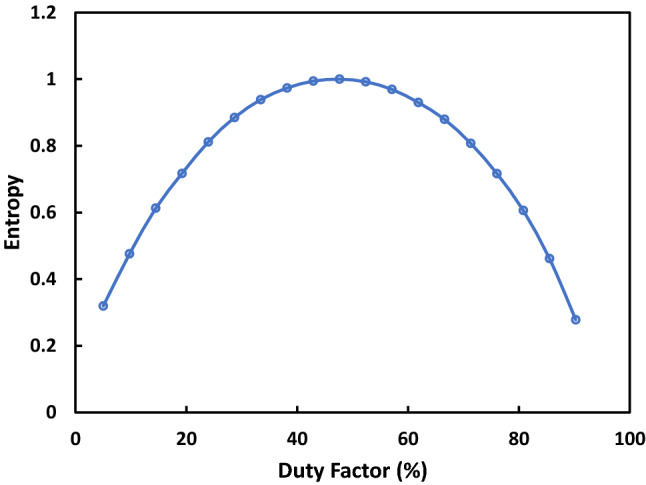
Shannon entropy of output bitstream when RTN signal is used to sample oscillators of different duty factors. Shannon entropy is used to measure the ratio of ‘0’ bits to ‘1’ bits. A Shannon entropy of ‘1’ indicates a 50% of any selected bit being ‘0’ or ‘1’. A Shannon entropy less than 1 is caused by a bias in the bitstream to either ‘0’ or ‘1’, indicating that the bitstream is not random. A 100 kb bitstream was generated at each data point to be tested. 9 NIST tests were done on these bitstreams to find the largest error from 50% duty factor that could pass. When the largest error that could pass 9 NIST tests was found, a 3 Mb bitstream was generated with that error to verify the results with all 15 NIST tests.

For the duty factor of the oscillator the key point is that it should be kept as close to 50% as possible. This is because a duty factor significantly different to 50% will result in a bias of the output bitstream to either ‘0’ or ‘1’. Figure 7 shows the relationship between duty factor of oscillator and the Shannon entropy of the output bitstream. Shannon entropy is used to check if there is any bias towards one value. For a system with two possible outputs, the ideal result for Shannon entropy is ‘1’. Any reduction from this represents a reduction in the randomness of the results. To find the maximum allowable error in the duty factor away from 50% the NIST test suite was used to test bitstreams generated from a simulated TRNG over a range of duty factors. It was found that the maximum variation away from 50% that would still pass all 15 NIST tests was 6%. If the oscillator has duty factor that is not ideal but has a consistent frequency greater than double Fosc_min_ the duty factor can be improved by use of an extra debias module. This module takes the unbalanced oscillator as the input and toggles the output every time a rising edge is detected in the input. This follows the debiasing scheme outlined in reference^39^.

### Bitstream analysis and NIST test results

**Figure 8:**
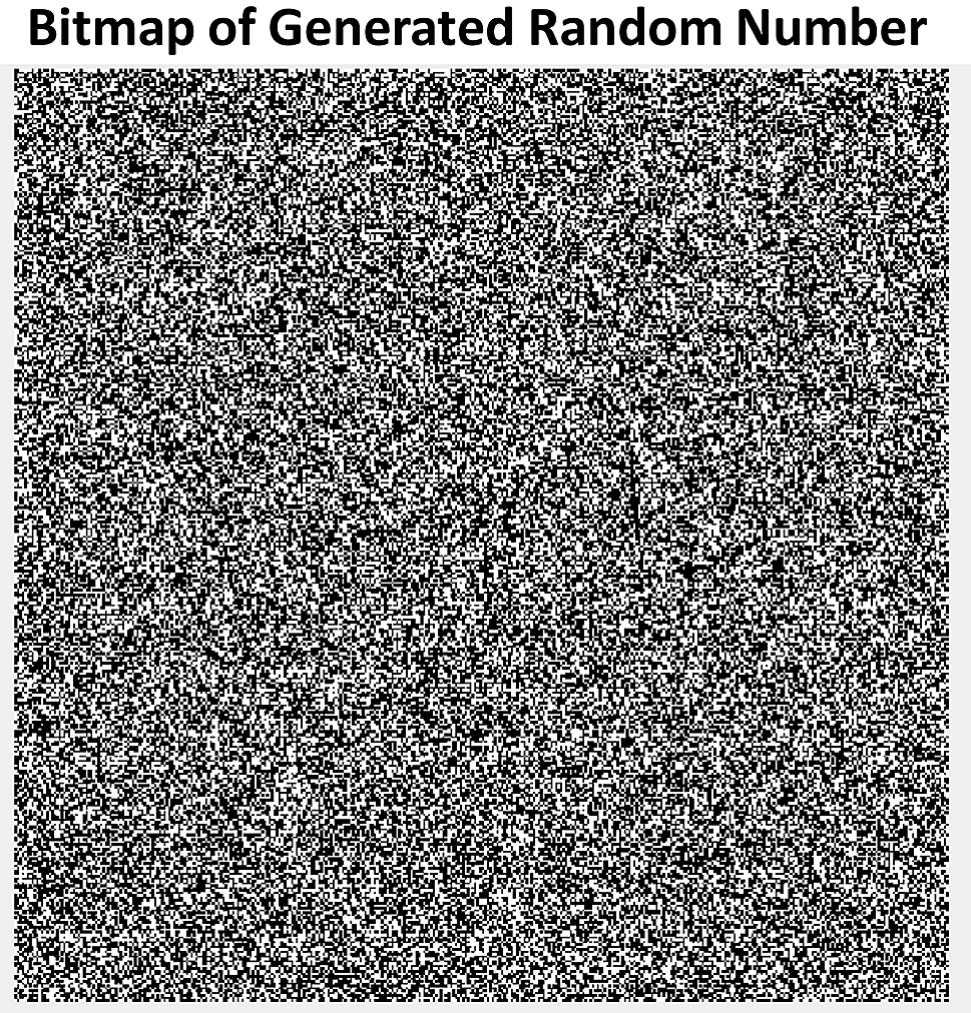
Bitmap showing the first 146 k bits of the generated bitstream from the TRNG proof of concept circuit. There are no clear repeating patterns, large blocks or lines of a single colour, and an even balance of black and white within the bitmap, which illustrates to the eye that the sequence is random, further supporting the results from the NIST test suite.

To verify the randomness of the generated bitstreams, they were transmitted to an external analysis PC. On the analysis PC, bitstreams were divided up into blocks whose sizes are defined by the guidelines provided in the instruction manual for the NIST test suite^41^ (see more details in the “Methods” section) and all 15 NIST tests were run. The significance value for our NIST tests was set to 0.01 which is the recommended value from NIST. For the tests excluding the non-overlapping template matching, random excursions, and random excursions variant, multiple blocks of random numbers are tested, and a proportion defined by NIST must pass the test for the bitstream to be considered random. For the three tests that don’t require multiple blocks tested, the individual P-value is used to determine success or failure. The bitstreams from our TRNG have passed all 15 NIST tests without post-processing as shown in Table 1. Our generated bitstream was also tested by using the DIEHARD test suite^47^, as detailed in the “Methods” section. The DIEHARD tests work on the same null-hypothesis theorem as the NIST tests, so that we kept the same significance value of 0.01 for determining P-value success or failure. The results of the DIEHARD tests are shown in Table 2. Our design was able to pass all of the available DIEHARD tests, giving a great confidence in the truly random nature of our generated bitstream. To further illustrate the randomness of our generated bitstream, a sample set of 146 k bits were used to create a bitmap in Fig. 8, where no clear patterns can be seen, confirming the randomness of the bitstream.

**Table 1:** NIST Test suite results for bitstream generated by our TRNG design with a significance value of 0.01.

Test ID	Name	Proportion	Pass/fail
T1	Monobit test	99/100	Pass
T2	Frequency tests within a block	100/100	Pass
T3	Cumulative sums test	96/100	Pass
T4	Run test	99/100	Pass
T5	Longest run of ones in a block	98/100	Pass
T6	Discrete fourier transform test	49/50	Pass
T7	Non-overlapping template matching	n/a	Pass
T8	Approximate entropy test	96/100	Pass
T9	Serial test	100/100	Pass
T10	Binary matrix rank	99/100	Pass
T11	Overlapping template matching	8/10	Pass
T12	Universal	8/10	Pass
T13	Linear complexity	10/10	Pass
T14	Random excursions	n/a	Pass
T15	Random excursions variant	n/a	Pass

**Table 2:** DIEHARD test results.

Test ID	Name	P-Value	Pass/Fail
T1	Birthday spacings test	0.87125	Pass
T2	Overlapping permutations	0.98398	Pass
T3	Rank of matrices	0.56833	Pass
T4	Bitstream test	0.72978	Pass
T5	Count-the-1’s stream test	0.36753	Pass
T6	Count-the-1’s byte test	0.22222	Pass
T7	Parking lot test	0.94059	Pass
T8	Minimum distance test	0.12461	Pass
T9	3D spheres test	0.12013	Pass
T10	Squeeze test	0.55082	Pass
T11	Overlapping sums test	0.16278	Pass
T12	Runs test	0.95088	Pass
T13	Craps test	0.35721	Pass
T14	Random excursions	n/a	Pass
T15	Random excursions variant	n/a	Pass

### Resistance to machine learning attacks

To test how our TRNG would resist machine learning attacks and further improve confidence in the security of the TRNG, we tested some of our generated bitstreams with an LSTM neural network. LSTM networks have been shown to be able to predict some random number generator outputs, even if the RNG passes all of the NIST tests^48^. We used 1 million generated bits for testing with the LSTM network (see details in the “Methods” section) and tried to predict future bits that will be generated by the TRNG. After training the LSTM network was only able to predicts the bits of our TRNG output with an accuracy of 49.81%, which is very close to the ideal value of 50% that could be achieved through random guessing. This shows that our design is resilient against machine learning based attacks and further increases confidence in the random nature of the output.

## Discussion

Previously proposed RTN based TRNGs could not extract the full entropy from multi-level or abnormal RTN. They rely upon finding a device from a large array that fits the ideal requirements of the TRNG^37^. These ideal devices can be very rare, resulting in large TRNG footprint as many devices have to be included in the array as well as complicated tuning and selection circuits to identify and select the ideal device. In our new design the ability to use devices that have multi-level or abnormal RTN vastly increases the chances that a device will be suitable for use in a TRNG. This means that the size of array required for potential entropy sources can be reduced to fewer than 10 devices. There is further benefit to using multi-level RTN in terms of output bit rate. In traditional designs where multi-level RTN might be present, the device is still usable if one trap is significantly larger than the other. In this case, only the entropy from this largest trap, however, was extracted. With our design, we can extract entropy from all of the traps. This allows us to utilise many more RTN events in a given time frame compared to a traditional design.

The new processing scheme proposed in this work gives us several key advantages over previous works. The first one is the removal of the complicated tuning of a sampling clock frequency. In traditional designs, the sampling clock frequency must be heavily correlated with the capture and emission times of the device being used as an entropy source in order to maintain a high output bitrate, while also avoiding oversampling of the entropy source. As the capture and emission times of a given device are unknown and can have a large device to device variation, the selection of a sampling clock frequency is complicated as it has to match a specific device before manufacture or the TRNG must have a complex self-tuning mechanism that will greatly increase design complexity and power consumption. With our proposed TRNG design the RTN events are used to do the sampling, so that no tuning is needed as long as the oscillator being sampled is running at a higher frequency than the RTN events. This sampling technique also makes our design immune to over-sampling attacks which has been known to be an issue for previous TRNGs^26^, where a higher frequency signal is imposed on top of the sampling clock to oversample the RTN signal and give long chains of ‘1’ or ‘0’. It is important to note that our design would be susceptible to under-sampling attacks which would give the same effect as oversampling for a traditional design. It is more difficult, however, to remove edges from the oscillator signal, without having direct access to the oscillator itself, than to add extra edges to a signal by simply applying an additional signal on top of it.

Another key advantage of the new processing scheme is that it removes the need for complicated post-processing. In traditional designs, Von Neumann or ORing post-processing were the most popular techniques to remove bias from the output^10^. These techniques require large complex circuits to implement and reduce TRNG throughput. More recently new post-processing techniques have been proposed that use a toggling output for each input rising edge from the entropy source^39^. This greatly reduces the circuit complexity, but it also greatly reduces the bitrate of the design. With our new technique no post-processing is required, as long as the sampling oscillator has a duty factor between 44% of 56%.

The current bitrate of our TRNG is limited to 19,200 bps by the requirements of the RS232 communication. For practical applications, this transmission circuit is not required so this limitation on the maximum bitrate would be removed. With the data transmission bitrate limit removed the next limiting factor is the slew rate of the op-amps used in the edge-to-pulse module. With the currently selected components the slew rate allows us to achieve a bitrate of ~ 10 Mbps. If the speed of the RTN exceeds the slew rate of the op-amps, then the differentiator will not complete its transition for each RTN event and will give an ambiguous output. This ambiguous output is maintained above the reference voltage of the comparator. As the input voltage to the comparator is always above the reference value, the TRNG will not generate random numbers. However, in our work the RTN found has not had time constants capable of generating bits at a rate higher than a few Mbps which is well below the maximum slew rate of the op-amps used.

The speed of the RTN events ultimately governs the bitrate of the TRNG. At the current bitrate limit of 19,200 bps it is very common to have RTN which is capable of generating bits at this rate. As the bitrate increases to a level that the rate of the RTN events becomes the limiting factor, there are several options available to increase the TRNG throughput. The first option is to find a device with faster RTN events. As mentioned earlier RTN event rate has a large device to device variation. Some devices may be capable of generating TRNGs with a bitrate in the Mbps range^49^ while other can only achieve a few kbps. In our measurements it is common to find devices that have traps whose time constants are in the microsecond range. Using one of these traps would allow for a maximum random bit generation rate of ~ 1 Mbps. In practice all TRNGs could be tested after manufacture and have their throughput rated accordingly so a consumer can select a TRNG with a suitable speed for their given application. Another option for increasing throughput is to use an AC RTN scheme for the voltages applied to the entropy source device. An AC RTN scheme has been shown to be capable of increasing the observed rate of RTN events by thousands of times^38^. The downside to using this AC RTN scheme is an increase in design complexity. The final option for increasing throughput is to have multiple devices used in parallel as the entropy source, doing this could massively increase the bitrate. As for the AC RTN, having multiple devices as the entropy source increases design complexity so it should be avoided if not necessary for a given application.

Another factor to consider for TRNG operation is the temperature effect on the TRNG. The key effect of temperature on the proposed TRNG is its effect upon the rate of RTN events. The effect of temperature on the capture and emission times of RTN traps has been studied in some detail^50^. It is known that typically an increase in temperature will decrease both the capture and emission times of an RTN trap. For our TRNG higher temperature will increase bitrate. In terms of security, these variations will not affect the randomness of the generated bitstream thanks to the RTN sampling oscillator scheme. The only time security that could be compromised is if the RTN is accelerated to such an extent that it surpasses Fosc_min_ and the RTN starts oversampling the oscillator. To avoid this, Fosc should be selected to be sufficiently above the maximum possible rate that RTN could be accelerated by raising temperature.

A further consideration for the parameters related to the entropy source is the voltage applied to it, namely Vg and Vd. For the entropy source devices used in this work the, the minimum applied Vg should be 0.42 V, which is close to threshold voltage and gives a sufficient drain current for measurement. The maximum Vg is 1.2 V. The Vd can be adjusted between 0.1 V and 0.5 V to achieve the optimal result. The effect of Vg and Vd on RTN traps has also been studied previously^46,51^. Typically, an increase in Vg will decrease the capture time and increase the emission time with a given Vd and the exact relationship being highly device dependant. For Vd variation with a given Vg, an increase in Vd will cause a decrease in emission time and an increase in capture time^52^, again with the exact relationship being highly device dependant. For low power applications it is advisable to use the lowest possible value for Vg and Vd as this will give the lowest current flow through the device, thereby reducing power consumption. It has also been reported that a low Vg will give the largest single trap impact on the Id within the device, making extraction of the RTN signal easier.

We now compare our design with some of the prominent TRNG designs previously proposed. The key figures for the discussed designs are shown in Table 3. Firstly, we look at the original RTN based TRNG proposed by Brederlow et al.^37^ The key improvements over this design include removing the post-processing circuit and thereby increasing design efficiency. As we use a much broader range of RTN types, it reduces the required size of device array.

**Table 3:** Comparison with Other TRNGs.

TRNG	Entropy source	Generation rate	Design area	Post processing	Verification
This work	Random telegraph noise	19.2 kbits/s^a^	N/A	None	Passes NIST and DIEHARD
Sanu 2015^15^	Metastability of inverters	162.5 Mbit/s	1088 μm^2^	XOR decorrelator and BIW extractor	Passes NIST
Figliolia 2015^53^	RTN with sigma delta converter	1–25 Mbit/s	2200 μm^2^	None	N/A
Matsumoto 2008^54^	Tunnelling in SiN device	0.3 Mbit/s	1200 μm^2^	None	Passes FIPS 140-2
Brederlow 2006^37^	Random telegraph noise	200 kbits/s	0.009 mm^2^	Von Neumann	Passes NIST
Chen 2015^40^	Random telegraph noise	0.25–5 Mbits/s	N/A	Von Neumann or bit truncation	Passes NIST
Jiang 2017^33^	Memristor write time	6 kbits/s	N/A	None	Passes NIST with reduced α value
Mohanty 2017^39^	Random telegraph noise	3 bits/s	N/A	None	Passes NIST

^a^Speed when limited by RS232 communication.

The design of Figliolia et al.^53^ does have potentially greater generation rate, but is significantly more complex and requires a lot more design area when implemented onto an IC. Also, Figliolia’s design was not tested using the DIEHARD or NIST test suite, so the randomness of the generated bitstream is not verified.

Chen et al.^40^ proposed a design that can either offer comparable or better bitrate than our design depending upon the type of device used and the level of confidence in the security that is required. At a similar security confidence, the bitrate will be similar to ours, if the RS232 limitation is removed and a single entropy source is utilised. Our design offers improvements over Chen’s design in areas of design complexity and efficiency of entropy extraction as we utilise more types of RTN. Chen’s design is only tested in a simulated environment, rather than with a practical circuit, so that there is more uncertainty in the real-world performance of their design when compared to ours.

The design by Mohanty et al.^39^ improves the efficiency when compared to other previous RTN based designs. Our proposed design further improves the efficiency. Mohanty removes the need for post-processing circuits through a balancing scheme. We also remove the post-processing circuits by using RTN to sample an oscillator. For multi-level RTN, Mohanty’s proposed design extracts the entropy from the slowest trap, whereas our design extracts the entropy from all RTN events. This improved entropy extraction is the primary reason why our design generates bits at a significantly higher rate as compared with Mohanty’s design.

Next, we compare our design with some of the most prominent non-RTN based TRNGs. The metastability based TRNG proposed by Sanu et al.^15^ can achieve bitrates higher than any of the RTN based TRNGs, but it is significantly more complex and requires tuning and processing circuits, making it suit high performance, high power applications. On the other hand, the simplicity and low power of our design fits better for applications such as remote IoT sensors or ID cards.

Matsumoto et al.^54^ proposed a design based on tunnelling in SiN devices. Its bitrate is lower than the bitrate of our design without the RS232 limitation and its area is even larger than that of Sanu et al.^15^. Matsumoto also mentions that the proposed design only works well with a specific type of device, whereas our CMOS-based design does not have this limitation.

The design proposed by Jiang et al.^33^ is based on the write time of memristors. Jiang’s design can achieve a bitrate that is comparable with our design when limited by the RS232 and would be significantly slower than our design with that limitation removed. Their design has a similar level of complexity as ours. Our design has the advantage that it is based on CMOS technologies, making it readily implementable.

In conclusion, we present an experimentally verified TRNG design, based on the trap event timing of multi-level RTN, using standard CMOS technology. The proposed design makes use of a new edge-to-pulse entropy extraction technique, which enables us to utilise multi-level RTN, abnormal RTN as well as traditional two level RTN, thereby improving on device selectivity and output bitrate. The design also introduces a new processing technique, which removes the need for any post-processing, the need for complicated tuning circuits for sampling frequency, as well as increasing resistance to oversampling attacks. The randomness of the proposed TRNG is verified by the 15 NIST Special Publication 800-22 randomness tests as well as the DIEHARD randomness tests. The proposed design also shows resilience against machine learning attacks. Selection of key parameters is discussed for optimising TRNG performance in terms of output bitrate and design complexity for different user requirements. The novel improvements presented in this work pave the way for a TRNG suitable for the low power, low area, and low-cost applications that are key requirements of IoT edge units.

## Methods

### Device information

The characterisation and TRNG testing was performed using both 22 nm FinFET NMOS devices provided by imec and 28 nm high-k metal gate PMOS devices provided by CSR. For the 22 nm devices the channel length was 28 nm and the width was 90 nm. For the 28 nm devices the channel length was 27 nm and the width was 90 nm.

### Device characterisation

All device characterisation was performed with a Keysight B1500 semiconductor analyser. An SMU was used to measure I–V signals and 2 WGFMU units were used to measure the constant Vg RTN. During TRNG operation, an Agilent Infiniium MSO8104A oscilloscope was used to observe the raw amplified RTN signal, as well as signals from several key points within the edge-to-pulse unit.

### TRNG proof of concept operation

For the proof of concept circuit, supply voltages were applied with Keysight E3632A DC power supplies, Id current with RTN events was amplified using a Femto DHPCA-100 amplifier, and a Xilinx Spartan 3 FPGA development board was used. The entropy source device was probed on a sample silicon wafer using a Cascade Summit probe station. FPGA programming was performed in the ISE design suite using a combination of custom and conventional VHDL blocks assembled in the visual programming environment. Videos of the TRNG proof of concept circuit in operation are available.

### NIST test suite testing

For each of the 15 NIST tests the recommended bitstream length and number of tested blocks was used, as well as any other recommended parameters specific for each test (see Supplementary Information for specific values). The significance value selected was 0.01, which is recommended in the NIST user manual. The test suite was run on a Linux machine running the Red Hat enterprise operation system. For the NIST results relating to the operation of the proof of concept circuit the bitstreams are saved onto the analysis PC after being transmitted from the FPGA in the form of a “.txt” file. For the NIST results relating to the oscillator parameter testing, the bitstreams are gained from Matlab simulations of the TRNG and are exported from Matlab into “.txt” files which are then tested in the NIST test suite on the Linux machine.

### DIEHARD tests

As the original version of the DIEHARD test suite is not readily available anymore, we made use of the Dieharder test suite constructed by Robert G. Brown^47^ for Duke University, which contains accurate reconstructions of each of the original DIEHARD tests. To perform the DIEHARD tests the binary bitstreams used for the NIST test were converted to a stream of 32-bit unsigned integers, as required by the “202” generator setting in the Dieharder test suite. 8 Mb of data was used for testing in the Dieharder suite. The length of the bitstreams used for each test were determined by the guidelines provided with the Dieharder test suite. The random excursions and random excursions variant tests were not included in the Dieharder test suite, however the tests are identical to the tests with the same name in the NIST test suite, so the NIST test results are shown. The Monkey tests (OPSO, OQSO and DNA) are listed as being unreliable so they were not used for the DIEHARD testing.

### LSTM neural network

The construction of the LSTM neural network was guided by the early works that used machine learning to try to crack random number generators^48,55,56^. Based on the principles outlined in these previous works, our LSTM network was constructed with an LSTM layer of 256 hidden units and a single hidden layer, connected to a fully connected layer. The training was performed with 1 million bits over 20 epochs. 100,000 predicted bits were generated and tested against 100,000 bits from the TRNG and the hamming distance between them was calculated. The hamming distance achieved was 0.4981.

### TRNG simulations for parameter testing

For the TRNG simulations that were used for exploration of the various oscillator parameters, Matlab was used to create and run the simulations. The RTN model within the simulated TRNG utilises the widely used model based on a first order Markov chain reported in reference^57^. The values used for the model input parameters are based on the values extracted from experimental characterisation of real transistors. The rest of the TRNG is simulated using Matlab script to generate the required oscillator signal and output bits from sampling the oscillator signal with the timing of the simulated RTN events.

## Supplementary information

Supplementary Information.

## Data Availability

All data used to support the findings of this paper are available from the corresponding author upon request.
