# Supplementary material for: Random-telegraph-noise-enabled true random number generator for hardware security
Source: Sci Rep. 2020 Oct 14;10:17210. doi: 10.1038/s41598-020-74351-y (PMC7560754; doi:10.1038/s41598-020-74351-y)
Supplement: Supplementary file 1 — Supplementary Information. [file 41598_2020_74351_MOESM1_ESM.docx]

**Random-Telegraph-Noise-enabled true random number generator for hardware security**

James Brown^1*^, Jianfu Zhang^1*^, Bo Zhou^1^, Mehzabeen Mehedi^1^, Pedro Freitas^1^, John Marsland^1^, Zhigang Ji^2*^

**NIST Test Parameters**

Significance value (α) = 0.01

Frequency monobit test – sequence length (n) = 100, number of sequences = 100

Frequency within a block test – sequence length (n) = 100, block size (M) = 20, number of sequences = 100

Runs test - sequence length (n) = 100, number of sequences = 100

Test for the Longest Run of Ones in a Block – sequence length (n) = 128, length of each block (M) = 8, number of sequences = 100

Binary matrix rank test – sequence length (n) = 38,912, number of sequences = 100

Discrete Fourier Transform (Spectral) Test – sequence length (n) = 1,000, number of sequences = 50

Non-overlapping Template Matching Test - bits of each template (m) = 9, length of the entire bit string under test (n) = 1,000

Overlapping Template Matching Test - sequence length (n) = 1,000,000, number of sequences = 10

Maurer’s “Universal Statistical” Test - sequence length (n) = 387,840, number of sequences = 10

Linear Complexity Test - sequence length (n) = 1,000,000, block size (M) = 500

Serial Test - sequence length (n) = 1,000, length in bits of each block (m) = 7, number of sequences = 100

Approximate Entropy Test - sequence length (n) = 1,000, length in bits of each block (m) = 4, number of sequences = 100

Cumulative Sums (Cusum) Test - sequence length (n) = 1,000, number of sequences = 100

Random Excursions Test - sequence length (n) = 1,000,000

Random Excursions Variant Test - sequence length (n) = 1,000,000

**LSTM Neural Network Layers**

sequenceInputLayer(numFeatures)

lstmLayer(numHiddenUnits)

fullyConnectedLayer(numResponses)

regressionLayer];

Max Epochs = 20
